# Docosahexaenoic acid (DHA): a modulator of microglia activity and dendritic spine morphology

**DOI:** 10.1186/s12974-015-0244-5

**Published:** 2015-02-22

**Authors:** Philip K-Y Chang, Armen Khatchadourian, Rebecca Anne McKinney, Dusica Maysinger

**Affiliations:** Department of Pharmacology and Therapeutics, McGill University, McIntyre Medical Building, Room 1314, 3655 Promenade Sir William Osler, Montreal, QC H3G 1Y6 Canada; Department of Pharmacology & Therapeutics, Bellini Life Science Complex, McGill University, Room 167, 3649 Promenade Sir-William-Osler, Montreal, QC H3G 0B1 Canada

**Keywords:** Docosahexaenoic acid, Lipid bodies, Mitochondria, Microglia, Neuroinflammation, Dendritic spines

## Abstract

**Background:**

Recent studies have revealed that excessive activation of microglia and inflammation-mediated neurotoxicity are implicated in the progression of several neurological disorders. In particular, chronic inflammation *in vivo* and exposure of cultured brain cells to lipopolysaccharide (LPS) *in vitro* can adversely change microglial morphology and function. This can have both direct and indirect effects on synaptic structures and functions. The integrity of dendritic spines, the postsynaptic component of excitatory synapses, dictates synaptic efficacy. Interestingly, dysgenesis of dendritic spines has been found in many neurological diseases associated with ω-3 polyunsaturated fatty acid (PUFA) deficiency and cognitive decline. In contrast, supplemented ω-3 PUFAs, such as docosahexaenoic acid (DHA), can partly correct spine defects. Hence, we hypothesize that DHA directly affects synaptic integrity and indirectly through neuron-glia interaction. Strong activation of microglia by LPS is accompanied by marked release of nitric oxide and formation of lipid bodies (LBs), both dynamic biomarkers of inflammation. Here we investigated direct effects of DHA on synaptic integrity and its indirect effects via microglia in the hippocampal CA1 region.

**Methods:**

Microglia (N9) and organotypic hippocampal slice cultures were exposed to the proinflammagen LPS (100 ng/ml) for 24 h. Biochemical and morphological markers of inflammation were investigated in microglia and CA1 regions of hippocampal slices. As biomarkers of hyperactive microglia, mitochondrial function, nitric oxide release and LBs (number, size, LB surface-associated proteins) were assessed. Changes in synaptic transmission of CA1 pyramidal cells were determined following LPS and DHA (25-50 μM) treatments by recording spontaneous AMPA-mediated miniature excitatory postsynaptic currents (mEPSCs).

**Results:**

Microglia responded to LPS stimulation with a significant decrease of mitochondrial function, increased nitric oxide production and an increase in the formation of large LBs. LPS treatment led to a significant reduction of dendritic spine densities and an increase in the AMPA-mediated mEPSC inter-event interval (IEI). DHA normalized the LPS-induced abnormalities in both neurons and microglia, as revealed by the restoration of synaptic structures and functions in hippocampal CA1 pyramidal neurons.

**Conclusion:**

Our findings indicate that DHA can prevent LPS-induced abnormalities (neuroinflammation) by reducing inflammatory biomarkers, thereby normalizing microglia activity and their effect on synaptic function.

**Electronic supplementary material:**

The online version of this article (doi:10.1186/s12974-015-0244-5) contains supplementary material, which is available to authorized users.

## Background

Chronic activation of inflammatory signaling cascades in the brain can play a critical role in the development of many neurodegenerative disorders [1]. This observation has led to investigations of the proinflammatory endotoxin lipopolysaccharide (LPS) and anti-inflammatory component of healthy diets rich in docosahexanoic acid (DHA) [2,3] to reduce the deleterious effects of inflammation [4,5]. DHA, a ω-3 polyunsaturated fatty acid (ω-3 PUFA), is abundant in the healthy brain, and it is enriched in the health-improving Mediterranean diets [2,6,7]. Randomized controlled studies with the nutritional supplement Souvenaid (containing DHA) suggest some functional improvements in a subpopulation of patients with the symptoms of mild Alzheimer’s disease [8,9]. However, there are still some inconsistencies in findings from clinical and pre-clinical studies with DHA as a therapy in neurological diseases, in particular Alzheimer’s disease [10]. The inconsistencies reported between the studies could be due to the inherent complexity of many neurological disorders, the diversity of populations used in different trials, different dosages and formulations, the stage of the disease when treatment began and other confounding factors [11,12]. In this article we set out to determine, in a simplified system, how DHA modulates inflammatory responses in N9 microglia and the hippocampus.

During inflammation, microglia, the resident immune cells in the brain, play a pivotal role in cerebral functions and act as major guardians of brains threatened by pathogens and the proinflammatory endotoxins they produce [13-16]. Recent studies revealed that microglia can also play a role in synaptic remodeling and plasticity in the healthy brain [17-20]. For example, microglia within the visual cortex can modify their association with dendritic spines in response to changes in visual sensory experience [21].

We selected to activate microglia by inducing inflammation using the most potent and commonly used proinflammatory stimulus, LPS, an endotoxin produced by gram-negative bacteria [22]. Hyperactive microglia produce and release inflammatory cytokines that can adversely affect synaptic structure [23,24]. Aside from known production of proinflammatory cytokines and nitric oxide, LPS-stimulated microglia form lipid bodies (LBs), organelles often found in macrophages exposed to infectious microorganisms or their endotoxins. LBs are organelles found in almost all eukaryotic cells, and their number and size vary in different cell types [25,26]. LBs were thought for many years to be storage sites for lipids; now they are beginning to emerge as dynamic organelles and signaling platforms affecting cell fate [27,28]. However the exact role of LBs in neural cell survival and maintenance of neural circuitries is unclear. Intriguingly, ω-3 PUFAs, such as DHA, can be temporarily stored in LBs and become available to alter excitatory synapse morphology and function. The mechanism of DHA effects on postsynaptic dendritic spines is currently unclear. To examine changes in synaptic structures we concentrated on dendritic spines, the postsynaptic structural components found on excitatory synapses of principal neurons in the brain [29-32]. These structures contain the molecular machineries necessary for synaptic transmission and can directly dictate the efficacy of neurocircuits [33,34]. Spines have the ability to respond to learning paradigms by changing their number and size [32,33,35]. Therefore, it has been proposed that these structures are the morphological correlates for cellular models of learning and memory [36,37]. In fact, dendritic spine dysgenesis is found in many neurological disorders accompanied with cognitive deficits [29,38-40].

In this study we sought to investigate how DHA modulates hyperactive microglia and synaptic function in hippocampal CA1 neurons. We paid particular attention to LBs and showed that they can play a protective role when neural cells are treated with DHA. The results also show that DHA prevented dendritic spine loss and an increase in AMPA-mediated miniature excitatory postsynaptic current (mEPSC) inter-event intervals (IEI) in hippocampal CA1 pyramidal neurons when exposed to inflammatory agents.

## Methods

### Organotypic hippocampal slice cultures and treatment

Organotypic hippocampal slice cultures were prepared as previously described [41]. Briefly, the slice cultures were prepared from P6-8 transgenic mice that expressed membrane-targeted eGFP under the Thy-1 promoter in a subpopulation of CA1 neurons. Following decapitation, hippocampi were dissected; 400-μm-thick transverse slices were made and adhered onto glass coverslips with chicken plasma clot (Cocalico Biologicals; Reamstown, PA, USA). Cultures were maintained in a roller drum incubator at 36°C for 3 weeks prior to experimentation to allow for maturation. Culture medium consisted of 25% heat-inactivated horse serum (Invitrogen GIBCO), 25% Hank’s balanced salt solution (Invitrogen GIBCO) and 50% Basal Medium Eagle (Invitrogen GIBCO) and was replaced weekly. Once ready, the cultures were incubated in serum-free medium overnight and treatments (LPS, 10 μg/ml; DHA, 25 μM; LPS + DHA) were applied for 24 h. In the case of LPS + DHA, DHA was prepared as DHA/BSA complex (detailed preparation method below) and was added to the cultures as a pretreatment supplementation overnight prior to the addition of LPS. All animal care and treatment procedures were performed in accordance with guidelines set by the Canadian Council on Animal Care and McGill Animal Welfare Committee.

### DHA/BSA complex preparation

DHA/BSA complex (hereafter referred to as DHA treatment) was prepared by adding 25 mg of DHA (Nu-Check Prep) to ~20 ml of fatty acid-free BSA solution (5% w/v, in KRBH buffer; Sigma-Aldrich). The DHA/BSA solution was incubated for 5 h at 37°C. After the incubation, the pH of the solution was adjusted to pH 7.4, and the solution was filtered through a 0.22-μm filter. The non-esterified DHA concentration in the solution was determined with the non-esterified fatty acid (NEFA) C method kit (Wako). The final molar ratio of DHA to BSA was approximately 6:1. Aliquots of the stock solution were flushed with argon to prevent oxidation of DHA and were stored at -80°C. In addition, the presence of BSA was controlled for in those cultures that did not receive the DHA treatment.

### Immunohistochemical staining of microglia lipid bodies and cytochrome c

Following treatment, hippocampal slice cultures were removed from the cover glass and fixed in 0.1 M phosphate buffer (PB) containing 4% paraformaldehyde overnight at 4°C (pH 7.4). After fixation, the cultures were washed in 0.1 M PB, permeablized in 0.4% Triton X-100 and blocked with 1.5% heat-inactivated horse serum overnight at 4°C. Primary antibodies were incubated for 5 days at 4°C in the permeablizing solution at a 1:400 dilution for ionized calcium-binding adapter molecule-1 (Iba-1) (Wako Chemicals USA, Inc., Richmond, VA, USA) and 1:400 dilution for perilipin-2/adipophilin (Plin-2/ADRP; PROGEN Biotechnik GmbH., Heidelberg Germany), 1:400 dilution for perilipin 2(ADRP; PROGEN Biotechnik GmbH., Heidelberg Germany) and 1:400 dilution for cytochrome *c* (BD Pharmigen, Mississauga, Ontario, Canada). Secondary antibodies, DyLight 549 and 649 (Jackson ImmunoResearch Laboratories Inc., West Grove, PA, USA), were prepared at 1:250 dilutions in 0.1 M PB containing 1.5% heat-inactivated horse serum and incubated overnight. Slices were then mounted with DAKO Fluorescent Mounting medium (Dako Canada, Mississauga, Ontario, Canada) onto microscope slides prior to imaging and subsequent blinded analysis.

### Confocal microscopy and quantification

Fluorescently labeled microglia and eGFP-expressing CA1 pyramidal neurons were imaged. For labeled microglia, the images were acquired using an upright Leica TCS SP2 confocal microscope (Leica Microsystems, Heidelberg, Germany) equipped with an HCX PL APOCHROMAT 63× NA 1.4 oil-immersion objective. Image stacks were collected at Z = 0.25 μm and averaged four times. Image stacks were deconvolved with Huygens Essentials software (Scientific Volume Imaging, Hilversum, The Netherlands) using a full maximum likelihood extrapolation algorithm. Three-dimensional volume rendering and LB size quantification were carried out with Imaris 7.0.0 software (Bitplane, Zurich, Switzerland). LBs were further categorized into small (diameter <0.5 μm), medium (diameter >0.5 μm and <1.0 μm) or large (diameter >1.0 μm) for their distribution comparisons using the *Spot* function in the Imaris software.

For dendritic spine analysis, the images were acquired using a Zeiss LSM 710 confocal microscope (Carl Zeiss MicroImaging GmbH) with a W Plan-APOCHROMAT 63×/1.0 objective. Either eGFP-expressing secondary or tertiary dendritic branches from either apical or basal dendrites were imaged. Dendritic spine quantification was also carried out using Imaris software (Bitplane). Dendritic spines were classified into three main subtypes, stubby, mushroom and thin-type spines, using previously established methods based on the morphological measurements of the spine head and neck diameters [42].

### Mitochondrial functional analysis with JC-1

Following treatment, the slice cultures were examined for mitochondrial depolarization using the JC-1 dye (5,5′,6,6′-tetrachloro-1,1′,3,3′-tetraethylbenzimidazolylcarbocyanine iodide; Life Technologies, Burlington, Ontario, Canada), a positively charged cationic dye that exhibits membrane potential-dependent accumulation in mitochondria indicated by a fluorescence emission shift from red (~590 nm) to green (~525 nm). Slice cultures were incubated with 15 μM of JC-1 for 45 min at 36°C. Following incubation, slices were transferred to a temperature-controlled perfusion chamber mounted on an upright microscope (Carl Zeiss MicroImaging GmbH) with a W Plan-APOCHROMAT 63×/1.0 objective and continuously perfused with Tyrode solution containing (in mM): NaCl, 137; KCl, 2.7; CaCl_2_, 2.5; MgCl_2_, 2; NaHCO_3_, 11.6; NaH_2_PO_4_, 0.4; glucose, 5.6 (pH 7.4). Images were acquired at Z = 0.25 μm and averaged four times. Image stacks were deconvolved with Huygens Essentials (Scientific Volume Imaging) and quantified using Imaris (Bitplane). Mean fluorescence intensity of the image stacks were measured separately in both the green and red channels.

### Electrophysiological recordings

Slices were transferred into a temperature-controlled chamber (30°C) mounted on an upright microscope (DM LFSA, Leica Microsystems) and continuously perfused with Tyrode solution. Patch and field recording electrodes were pulled from borosilicate glass (GC150TC; Clark Instruments, UK). All electrophysiological recordings were made using an Axopatch 200A amplifier (Molecular Devices, Sunnyvale, CA, USA). Littermate and sister cultures were used to eliminate potential inter-experimental discrepancies. To record AMPA-mediated mEPSC, whole-cell voltage-clamp recordings from CA1 pyramidal neurons were obtained at 30°C using electrodes with resistances of 4-5 MΩ and filled with intracellular solution containing (in mM): K-gluconate, 120; EGTA, 1; HEPES, 10; Mg-ATP, 5; Na-GTP 0.5; NaCl, 5; KCl, 5; phosphocreatine, 10; 295 mOsm; pH adjusted with KOH to 7.3. AMPA-mediated mEPSCs were recorded with 1 μM tetrodotoxin (TTX), 15 μM CPP, 100 μM picrotoxin and 1 μM CGP55845 in the external Tyrode’s solution at -60 mV. Access resistance was monitored with brief test pulses at regular intervals (2-3 min) throughout the experiment. Access resistance was usually 10-13 MΩ, and data were discarded if the resistance deviated more than 20% through the course of the experiment. After the holding current had stabilized, data were recorded at a sampling frequency of 10 kHz and filtered at 2 kHz for 10 to 15 min. All mEPSCs were detected offline using the Mini Analysis Software (Synaptosoft, Decatur, GA, USA). The amplitude threshold for mEPSC detection was set at four times the root-mean-square value of a visually event-free recording period. Every recording was selected for blinded analysis of IEI and amplitude. The data obtained were then used to plot cumulative histograms with an equal contribution from every cell. For statistical analysis, data were averaged for every single cell.

### Cell culture and treatments

Murine immortalized microglia (N9) cells obtained from Dr. Seguela, (Montreal Neurological Institute) were seeded in Iscove’s Modified Dulbeco’s Medium (IMDM; Gibco) containing 5% fetal bovine serum (Gibco) and 1% penicillin-streptomycin (Gibco). Cells were maintained at 37°C in a humidified atmosphere containing 5% CO_2_. Adherent cells were treated with LPS (100 ng/ml; Sigma-Aldrich), DHA (50 μM; Nu-Chek Prep).

### Measurement of mitochondrial metabolic activity in microglia N9 cells

Mitochondrial metabolic activity in N9-treated cells was assessed by measuring the extent of thiazolyl blue tetrazolium bromide (MTT) (Sigma-Aldrich) reduction to formazan. Cells were seeded in 24-well plates (Sarstedt) at a density of 5 × 10^4^ cells/well. Following treatments with DHA, or LPS or LPS and DHA for 24 h, media were removed and replaced with serum-free media containing MTT (0.5 mg/ml). After 30 min of incubation at 37°C, media were aspirated from each well, and formazan crystals were dissolved in dimethylsulfoxide (DMSO) (Sigma-Aldrich). The formazan obtained from the reduction of MTT was measured by using a Benchmark microplate reader (Bio-Rad) at 595 nm. All measurements were done in triplicates, in three or more independent experiments.

### Lipid body (LB) labeling

LBs in N9 cells fixed with 4% paraformaldehyde were visualized with the neutral lipid staining fluorophores BODIPY 493/503 (4,4-difluoro-1,3,5,7,8-pentamethyl-4-bora-3a,4a-diaza-s-indacene; Invitrogen) or HCS LipidTOX Deep Red (Invitrogen). Stock solution of BODIPY 493/503 (4 mM) was made by dissolving the powder in DMSO. Cells were incubated with BODIPY 493/503 (20 μM, in PBS, 10 min) or with HCS LipidTOX (1:200 in PBS, 30 min). Cells were washed with PBS prior to confocal imaging.

### Double labeling of mitochondria and LBs

Live microglial cells were incubated with Mitotracker Deep Red 633 (500 nM) for 3 min at 37°C. After a wash with sterile PBS, cells were incubated with BODIPY 493/503 (prepared in cultured media, 20 μM) for 10 min at 37°C. Cells were washed with PBS and then incubated in culture media during the imaging session.

### Confocal microscopy of N9 cell cultures

Images were acquired with a Zeiss LSM 510 NLO inverted confocal microscope using a Plan Achromat 63× 1.4 N.A. Oil DIC objective. Microglial N9 cells were seeded at a density of 1 × 10^4^ cells/well in confocal chamber slides (Lab-Tek, Nalge Nunc International) or on coverslips at a density of 1.5 × 10^4^ cells/well. Coverslips were mounted on glass microscope slides (Fisher Scientific) using glycerol-free mounting media Vectashield H-1000 (Vector) and were sealed around the perimeter with clear nail polish. Images of BODIPY 493/503-labeled LBs were acquired by using the argon 488-nm excitation laser. Imaging of Mitotracker Deep Red 633-labeled mitochondria was done using an HeNe 633-nm excitation laser. Alexa Fluor 594 was detected using an HeNe 543 excitation laser. All images were acquired at a resolution of 1,024 × 1,024 pixels and a zoom factor of at least 1.5. Z-stack images consisted of 15 to 20 optical sections and were taken at intervals of 0.3 μm.

### Analysis of LB volume and number in N9 cell cultures

Confocal images of fluorescently labeled LBs were analyzed using the Imaris software (Bitplane), similar to that described for the organotypic slice cultures.

### Western blot analysis

Whole cell extracts were made by lysing the N9 cells in NP-40 buffer (50 mM Tris pH 8.0, 137 mM NaCl, 1% NP-40, 10% glycerol) and were supplemented with a complete protease inhibitor cocktail (1 tablet per 25 ml of lysis buffer; Roche Applied Science) and with phosphatase inhibitors sodium orthovanadate (1 mM) and sodium fluoride (1 mM). Cell lysates were boiled in 6 × sample buffer (12% SDS, 30% glycerol, 0.2% bromophenol blue, 12% 2-mercaptoethanol, 0.375 M Tris HCl pH 6.8) at a 5:1 (cell lysate:sample buffer) ratio for 5 min, and proteins were resolved by SDS-PAGE and then transferred (1 h, 100 V, in ice-cold transfer buffer) to nitrocellulose membranes (Hybond, Amersham Bioscience). Blocking of the membranes was performed by incubating them in 5% milk or BSA (in TBS-T). Membranes were incubated with primary antibodies to perilipin-2 (ADRP) (1:500) (guinea pig polyclonal, Fitzgerald Ind. 20R-AP002), perilipin-3 (TIP47) (1:500) (guinea pig polyclonal, Progen GP30), p38 (rabbit polyclonal) (1:500) (Santa Cruz, sc-535), phospho-p38 (rabbit monoclonal) (1:1,000) (Cell Signaling #9215) and actin (mouse monoclonal) (1:1,000) (Millipore, MAB1501R) overnight at 4°C. Following incubation with primary antibodies, membranes were incubated with horseradish peroxidase-linked secondary antibodies anti-rabbit IgG (Amersham Biosciences, NA 934), anti-guinea pig IgG (Sigma, A5545) and anti-mouse IgG (GE Healthcare, NXA931). The binding of the secondary antibody to the primary antibody was visualized by using an ECL Plus detection kit and HyBlot autoradiography films (Denville). Films were scanned (gray scale at 16 bits), and relative intensities of the immunoreactive bands were analyzed using the gel analyzer tool in the Image J (1.42) software.

### Nitric oxide release

Nitric oxide release was measured using Griess reagent (Sigma) according to the protocol provided by the supplier. Briefly, following treatment, culture media were collected, and Griess reagent was added and incubated at room temperature for 15-20 min. After incubation, absorbance of the nitrite produced was measured at 540 nm using a spectrophotometer. All experiments were performed twice, and measurements were made in triplicate.

### Statistical analysis

All experiments were performed at least twice, and all samples were analyzed in triplicate. Data are expressed as mean ± SEM and analyzed by ANOVA using the Tukey and Dunnett post hoc test for multiple comparisons. Significant differences are indicated by *p < 0.05, **p < 0.01 and ***p < 0.001.

## Results

**Figure 1:**
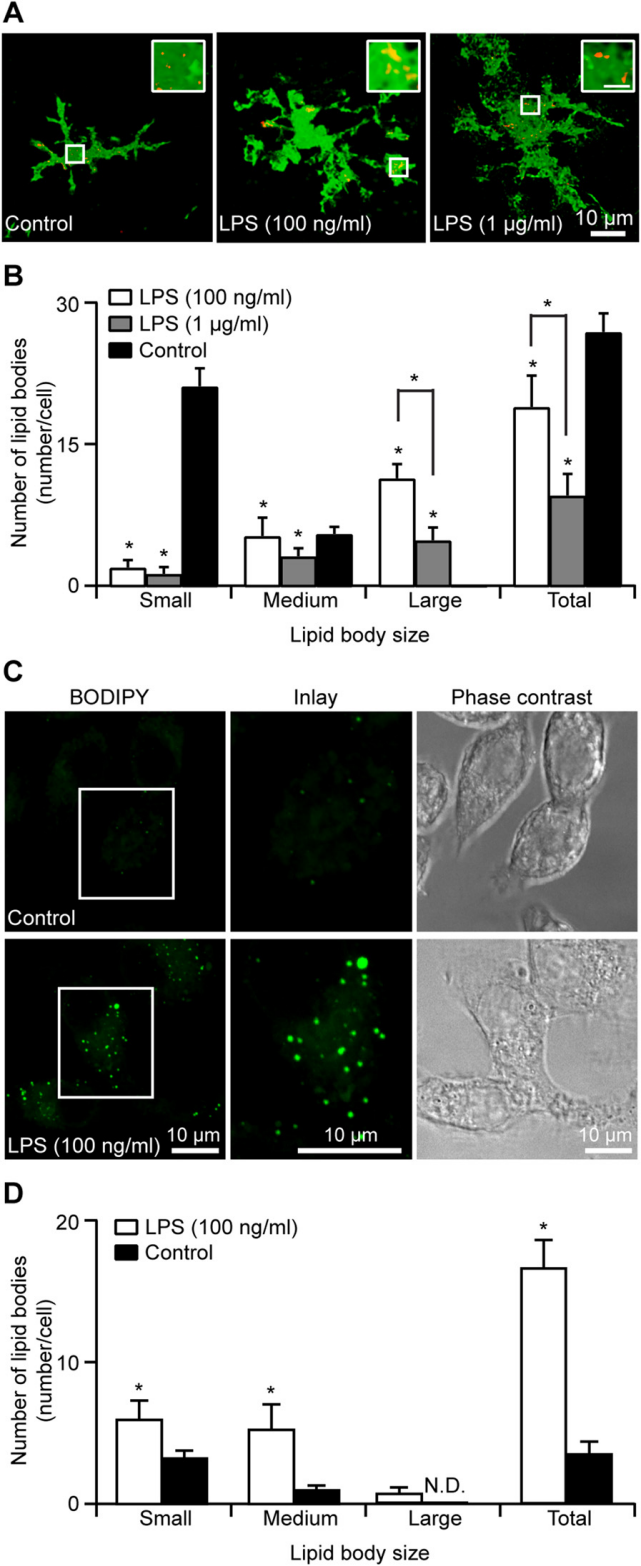
**LPS treatment alters LB number, increases LB size and causes microglia hypertrophy in organotypic hippocampal slice cultures and N9 microglial cells. A** Examples of LB formation in microglia from control and treated slices. In the presence of LPS (100 ng/ml or 1 μg/ml), more larger (>1.0 μm) LBs are present compared to control conditions. Microglial morphologies are also more ramified and appear to be more activated in the LPS-treated cultures. *Scale bar* 10 μm. *Green*, Iba-I. *Red*, ADRP. **B** LB density under various treatments with various LB sizes in microglia. *p < 0.05. **C** Examples of LB formation in N9 microglial cells showing that LPS treatment altered LB distribution. Following LPS treatment, there is an increase in LB formation. **D** LB densities and sizes following LPS treatment, showing that there is an increase in the large-sized LBs. *p < 0.05.

In order to establish a working model of neuroinflammation, we used the microglial activator LPS, a commonly used proinflammatory agent. We optimized the concentration of LPS for inflammation by testing a range of LPS concentrations in N9 cultures for 24 h (Additional file 1: Figure S1A). Inflammation was assessed by the degree of microglia activation by measuring nitrite (an indicator of nitric oxide release). We found a highly significant increase in nitric oxide release with 100 ng/ml LPS without any further increase with higher (1 μg/ml) LPS concentrations (Additional file 1: Figure S1A; control: 3.84 ± 0.26 μM; 100 ng/ml: 17.24 ± 0.86 μM; 1 μg/ml: 17.51 ± 0.44 μM; 10 μg/ml: 18.39 ± 0.34 μM; *p* < 0.05). Based on these findings, we chose to use either a low (100 ng/ml) or high (1 μg/ml) concentration of LPS to examine the formation of LBs after 24 h in both organotypic hippocampal slice cultures and N9 microglia cultures (Figure 1).

Next, we wanted to assess whether LPS induces changes in the activation of microglia by immunostaining of the biomarker for microglia activation, Iba-1. We revealed hypertrophic microglial morphology following LPS treatment at both high (1 μg/ml) and low (100 ng/ml) concentrations in organotypic hippocampal slice cultures, which contain both neurons and glia cells *in situ* (Figure 1A). Furthermore, in the same organotypic hippocampal culture system, both concentrations of LPS decreased the total number of LBs [control: 27.50 ± 2.01 LBs/cell, *n* = 13 cells from 8 slices; LPS (100 ng/ml): 19.4 ± 3.37 LBs/cell, *n* = 21 cells from 11 slices; LPS (1 μg/ml): 9.91 ± 2.26 LBs/cell, *n* = 11 cells from 6 slices]. When the size of LBs was examined, there was a decreased number of small (<0.5 μm) LBs in microglia [control: 21.60 ± 1.93 LBs/cell; LPS (100 ng/ml): 2.14 ± 0.81 LBs/cell; LPS (1 μg/ml): 1.45 ± 0.72 LBs/cell] and an increased number of large (>1.0 μm) LBs [control: 0.07 ± 0.07 LBs/cell; LPS (100 ng/ml): 11.71 ± 1.50 LBs/cell; LPS (1 μg/ml): 5.09 ± 1.33 LBs/cell]. Similarly, LPS treatments of N9 microglia increased the number of large LBs (Figure 1C-E; control: 3.62 ± 0.87 LBs/cell; LPS: 16.43 ± 2.01 LBs/cell; *p* < 0.05). These findings suggest that formation of the larger sized LBs is a hallmark of microglial inflammatory response in either microglia in the absence of neurons or in intact slice cultures containing both neurons and glia *in situ*.

**Figure 2:**
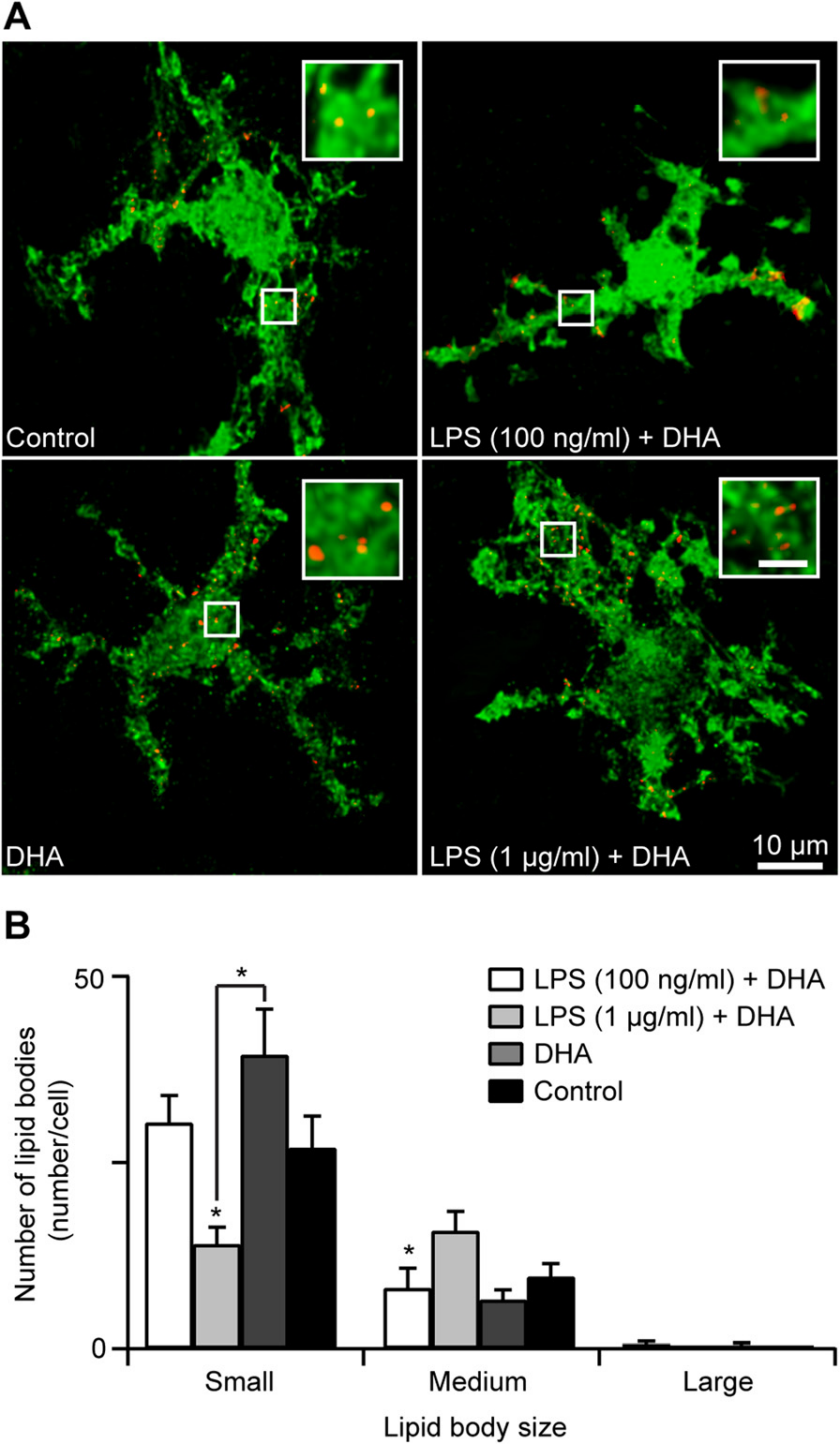
**DHA promotes small LB formation and normalizes LPS-induced microglial hypertrophy. A** Examples of activated microglial morphologies and LB formation from control and treated organotypic hippocampal slice cultures. In the presence of DHA, more small (<0.5 μm) LBs are present compared to other conditions. In LPS-treated cultures, the activated microglia contain fewer LBs compared to others. *Arrows* indicate small LBs; triangles indicate larger LBs. *Scale bar* 10 μm. *Green*, Iba-I. *Red*, ADRP. **B** LB density under various treatments with various LB sizes in microglia. *p < 0.05.

**Figure 3:**
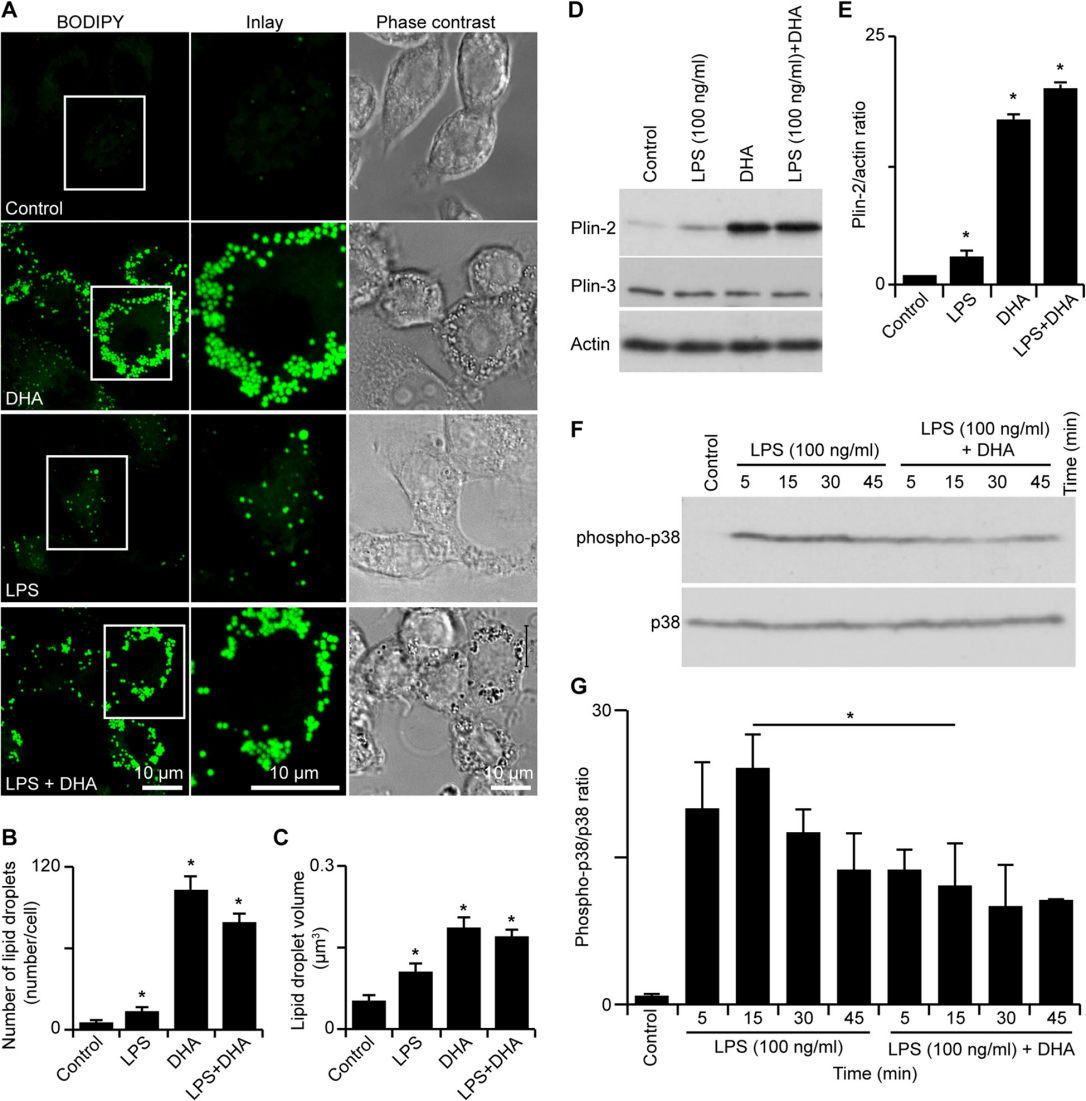
**DHA promotes small LB formation in N9 microglial cultures and normalizes LPS-induced p38 phosphorylation. A** Examples of LB formation from control and treated N9 microglial cultures. In the presence of DHA, more small (<0.5 μm) LBs are present compared to other conditions. LPS-treated culture microglial cells have fewer LBs compared to others. Scale bar 10 μm. **B** LB densities under different treatments. *p < 0.05. **C** LB volumes under different treatments. *p < 0.05. **D** Western blot analysis of Plin-2 and -3 following treatment of LPS and/or DHA. There was an increase in Plin-2 levels following LPS and DHA treatment. However, DHA greatly increased Plin-2 even in the presence of LPS. **E** Plin-2 and -3 ratios under various treatments. *p < 0.05. **F** Western blot analysis of p38 phosphorylation following LPS and DHA treatment. LPS treatment increases the p38 phosphorylation dramatically after a 5-min treatment. The phosphorylated p38 level reached a maximum 15 min following the addition of LPS. DHA treatment prevented this acute increase in p38 phosphorylation. **G** Ratios of phosphorylated versus unphosphorylated p38 levels as revealed by Western blot analysis following different treatments. *p < 0.05.

As it has been suggested previously that DHA and its derivatives have protective effects against inflammation, we examined whether the addition of DHA (25 μM) to organotypic slice cultures overnight (~16 h) prior to LPS treatment could prevent the LPS-induced changes of microglial morphology. We found that DHA alone did not change the appearance of microglia in the slice cultures when compared to control slices (Figure 2A). Interestingly, in those slices that were treated with DHA and 100 ng/ml of LPS, there was no observed microglial hypertrophy as seen with LPS treatment alone (Figure 2A). However, at higher concentrations of LPS (1 μg/ml), the addition of DHA did not prevent microglia from becoming hypertrophic in the presence of LPS (Figure 2A). This suggested that while DHA may have a protective role against lower grade neuroinflammation, this effect did not appear to be extended into more severe inflammation. We also investigated whether DHA supplementation can lead to a change in the number and size of LBs during neuroinflammatory response induced by LPS at the two concentrations. When we analyzed LB distributions in the labeled microglia, we found that the addition of DHA returned total LB numbers to the control level in both low [Figure 3B; control: 36.80 ± 3.98 LBs/cell, *n* = 18 cells from 10 slices; DHA: 46.40 ± 6.33 LB/cell; *n* = 20 cells from 10 slices; LPS (100 ng/ml) + DHA: 39.10 ± 4.51 LBs/cell, *n* = 21 cells from 11 slices] and high concentrations of LPS treatment [Figure 2B; LPS (1 μg/ml) + DHA: 30.11 ± 2.26 LBs/cell, *n* = 19 cells from 9 slices]. Upon closer examination of the different-sized LBs, we found that DHA prevented the appearance of the large (>1.0 μm) LBs that were seen after low [Figure 2B; control: 0.017 ± 0.090 LBs/cell; DHA: 0.00 ± 0.00 LB/cell; LPS (100 ng/ml) + DHA: 0.00 ± 0.00 LBs/cell] and high LPS treatments [Figure 3B; LPS (1 μg/ml) + DHA: 0.21 ± 0.034 LBs/cell]. There was also an increase in small-sized LBs (<0.5 μm), comparable to that of the control in DHA and LPS (100 ng/ml) + DHA-treated cultures [Figure 3B; control: 27.00 ± 4.19 LBs/cell; DHA: 39.45 ± 6.13 LB/cell; LPS (100 ng/ml) + DHA: 30.30 ± 3.63 LBs/cell]. However, in the 1 μg/ml LPS treatment, there were fewer small [Figure 2B; LPS (1 μg/ml) + DHA: 14.03 ± 2.27 LBs/cell] and more medium-sized LBs (>0.5 μm and < 1.0 μm) in microglia [Figure 3B; control: 9.67 ± 1.72 LBs/cell; DHA: 6.55 ± 1.32 LB/cell; LPS (100 ng/ml) + DHA: 8.10 ± 2.65 LBs/cell; LPS (1 μg/ml) + DHA: 15.84 ± 2.56 LBs/cell]. This suggested that DHA prevented the LPS-induced changes in LB and microglia morphology after exposure to lower concentrations of LPS. Interestingly, at the higher LPS concentration, DHA did not prevent the hypertrophic microglia, but did prevent the formation of large-sized LB formation.

In order to establish how the addition of DHA could prevent changes in microglia and LBs, we examined the impact of adding DHA (25 μM) to microglial N9 cultures prior to the treatment with LPS using further biochemical analysis to ensure that we were only measuring the biochemical changes found within microglial cells and not other cell types typically found in slice cultures, such as neurons and astrocytes. We found that although both LPS and DHA significantly increased the number of LBs in microglial cells, the increase was much more pronounced in cells exposed to DHA (~37 fold), while there was only a modest increase following LPS alone (~5 fold) treatment (Figure 3A-C). We also found that the addition of LPS to cells exposed to DHA significantly reduced the LB number/cell (*p* < 0.05) compared to cells treated with DHA alone (Figure 3A and B). Our reconstruction of the individual confocal Z-stack images of fluorescently labeled LBs and subsequent quantification of LB volumes revealed that an exposure of microglial cultures to LPS led to a significant increase in the average volume of LBs (control: 0.05 ± 0.011 μm^3^; DHA: 0.183 ± 0.02 μm^3^; LPS: 0.104 ± 0.016 μm^3^, *p* < 0.01). Treatment with LPS may have slightly reduced the relative abundance of small LBs (-14.9%; *p* > 0.05) and concomitantly increased the proportion of medium-sized LBs (+13.2%; *p* > 0.05) in microglial cells, but this was not significant.

Next, we investigated whether there were changes in LB-associated proteins essential for LB stability, growth and proliferation, namely perilipin-2 (ADRP) and perilipin-3 (TIP47) [43,44]. We performed Western blotting to compare how LPS and DHA influenced the levels of perilipin-2 and -3 in microglial cells (Figure 3D and E). Exposure of microglia to LPS alone led to a rise in the level of perilipin-2 associated with the accumulation of LBs [45,46]. DHA alone significantly increased the level of perilipin-2 (~17 fold; *p* < 0.001); this increase was considerably higher than that observed in microglial cells exposed to LPS alone (~3 fold; *p* < 0.05). In contrast, neither DHA nor LPS caused a significant change in the level of perilipin-3 in microglia. We also investigated how LPS influenced the extent of p38 kinase phosphorylation, as this kinase was implicated in LB generation. Western blot analysis revealed that exposure of microglial cells to LPS alone caused a transient increase in p38 phosphorylation that reached a maximum 15 min post LPS treatment (Figure 3F and G). Interestingly, DHA treatment of N9 microglia significantly reduced LPS-induced p38 activation (Figure 3F and G). DHA alone also induced p38 phosphorylation, but to a lesser extent than LPS (Additional file 1: Figure S2; *n* = 6; * *p* < 0.05). We also found that DHA in the presence of LPS led to a significant reduction in nitric oxide production, suggesting a reduction in iNOS induction (Additional file 1: Figure S1B; control, 0.67 ± 0.12 μM; LPS, 34.33 ± 0.49 μM; DHA, 0.94 ± 0.093 μM; LPS + DHA, 22.4 ± 0.51 μM. Control, *n* = 6; LPS, *n* = 6; DHA, *n* = 5; LPS + DHA, *n* = 5; * *p* < 0.05).

**Figure 4:**
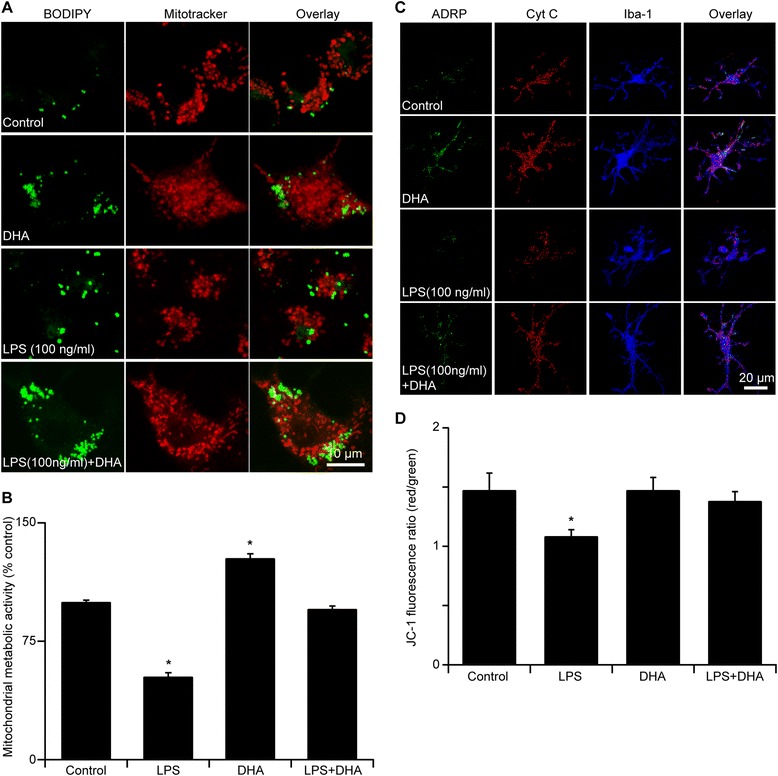
**DHA promotes the formation of small LBs, increases LB interaction with mitochondria and restores mitochondrial function in microglia from both N9 and hippocampal slice cultures. A** Fluorescent labeling of LBs and mitochondria in N9 cultures reveals that LBs are in close proximity with mitochondria under control and DHA-treated cultures but not in LPS-treated cultures. With DHA treatment, the LBs appear to form in large clusters. Scale bar 10 μm. **B** MTT assay detection of mitochondrial metabolic activity expressed as percentage of control-treated cultures. With 24 h of LPS treatment, there is a significant decrease in mitochondrial metabolic activity. With the addition of DHA alone, there is an increase in mitochondrial metabolic activity compared to control cultures. Lastly, when the cultures are treated with both LPS and DHA, the mitochondrial metabolic activity is restored to 100% of control. *p < 0.05. **C** Fluorescent labeling of LBs, mitochondria and activated microglia in hippocampal slice cultures revealed that LBs are in close proximity with mitochondria under control and DHA-treated cultures but not in LPS-treated cultures. With DHA treatment, the LBs appear to form in large clusters, similar to the findings reported in N9 cultures. Scale bar 20 μm. **D** JC-1 staining of mitochondrial membrane depolarization can be used as readout of mitochondrial health seen by a shift in the red/green fluorescence ratio in hippocampal slice cultures. The 24-h treatment with LPS significantly decreases the red/green ratio, an indication of mitochondrial depolarization and poor mitochondrial health. With the addition of DHA, the red/green fluorescence ratio is restored to control level. *p < 0.05.

LBs have been previously shown to form membrane contact sites with different organelles, including mitochondria [47,48]. The substantial changes in the number, volume and size distribution of LBs that we observed in microglial cells exposed to LPS and to DHA, or to their mixture, prompted us to examine possible LB-mitochondria contacts. Using confocal microscopy, we found that LBs and mitochondria are often found close to each other in microglial cells exposed to DHA or to DHA and LPS combined, but not to LPS alone. This was seen in both pure microglial and organotypic hippocampal slice cultures (Figure 4A and C). Smaller LBs may be more mobile and presumably more effective in providing energy to mitochondria. DHA-induced LB in N9 microglia restored the defective mitochondrial metabolism caused by LPS (Figure 4B; control: 100.00 ± 1.10%; LPS + DHA: 95.5 ± 1.80%; *p* > 0.05; Figure 4B; LPS: 52.70 ± 2.40%; *p* < 0.05). In support of this finding in N9 microglia, JC-1 staining for mitochondrial membrane potential in CA1 region of organotypic hippocampal slice cultures revealed that DHA treatment also restored the mitochondrial deficits caused by LPS treatment (Figure 4D; control: 1.48 ± 0.14; LPS: 1.09 ± 0.055; DHA: 1.48 ± 0.11; LPS + DHA: 1.38 ± 0.078; * *p* < 0.05; *n* = 12-15 image stacks from 4-5 cultures from each condition).

**Figure 5:**
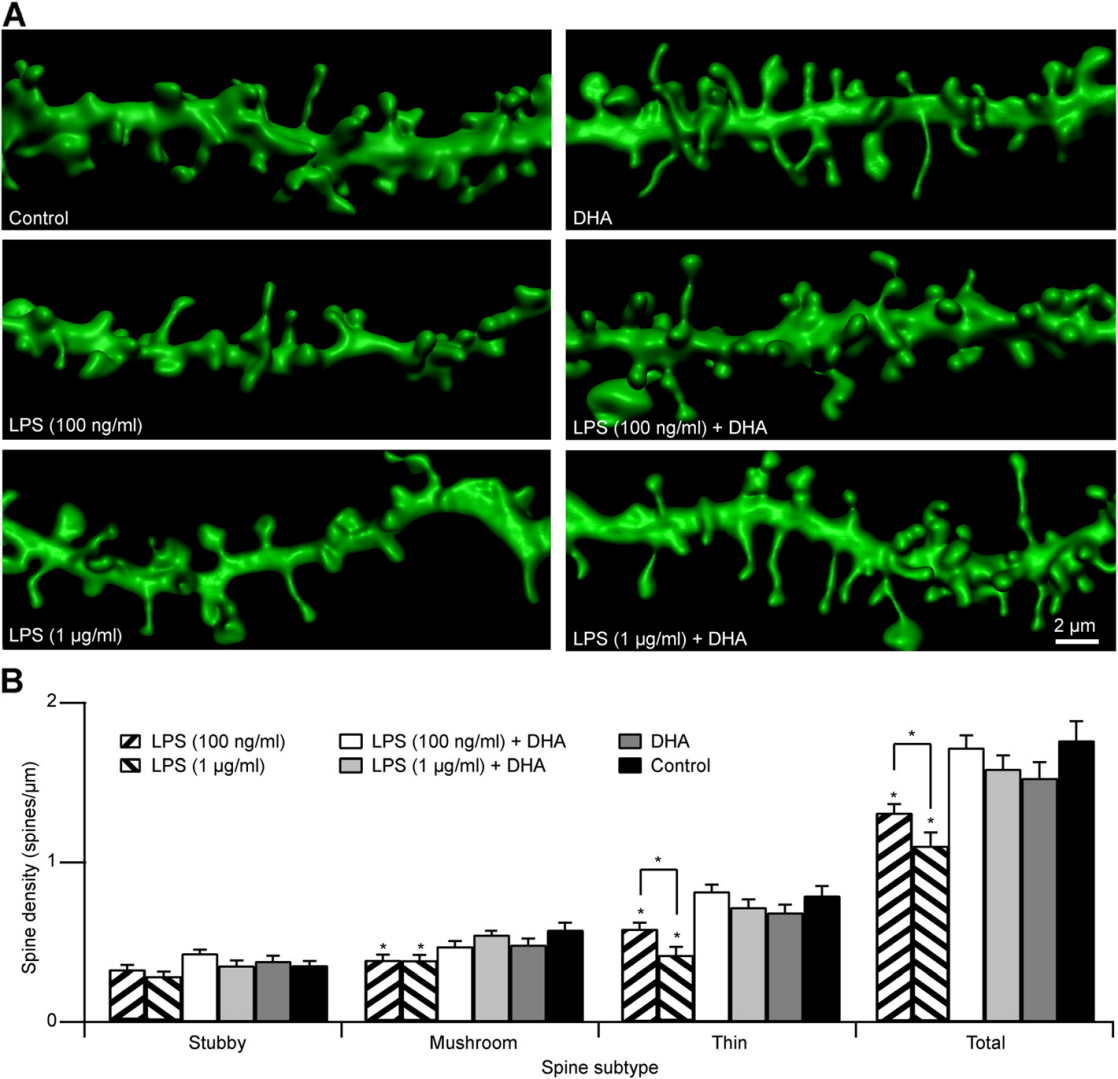
**LPS treatment decreases dendritic spine densities of CA1 pyramidal neurons in organotypic hippocampal slice cultures that are prevented by DHA. A** Examples of dendrites segments from CA1 pyramidal neurons showing control-, LPS-, DHA- and LPS + DHA-treated dendritic spine morphologies. In the presence of LPS, there is a decrease in spine densities. Higher LPS concentration (1 μg/ml) causes a more pronounced spine decrease compared to lower concentration of LPS (100 ng/ml). With DHA alone, there is no difference in dendritic spine morphologies compared to control. If the LPS-treated cultures are pre-incubated with DHA, no spine loss is observed. Lower (100 ng/ml) or higher LPS (1 μg/ml) concentrations do not differ in their response to DHA pre-treatment. Scale bar 2 μm. **B** Dendritic spine density under various treatments in CA1 pyramidal neurons. *p < 0.05.

Since microglia activation and neuroinflammation have been implicated in various neurological disorders, we next wanted to assess the impact of LPS treatment on synaptic structure by examining dendritic spines as their dysgenesis has been shown in many neuropathologies. As dendritic spines are the integral postsynaptic components of functional excitatory synaptic circuits, any compromise in their structures can negatively impact the normal physiology of the brain. With both low (100 ng/ml) or high (1 μg/ml) LPS concentrations, we found a significant decrease in total dendritic spine densities in CA1 pyramidal cells in hippocampal slice cultures [Figure 5; control: 1.67 ± 0.11 spines/μm, *n* = 25 cells, 1-2; dendritic segment of 20-50 μm per cell; LPS (100 ng/ml): 1.31 ± 0.056 spines/μm, *n* = 16 cells; LPS (1 μg/ml): 1.10 ± 0.085 spines/μm, *n* = 21 cells]. The decrease in spine numbers could be attributed to the reduction of both larger, mushroom- [Figure 5; control: 0.63 ± 0.062 spines/μm; LPS (100 ng/ml): 0.40 ± 0.037 spines/μm; LPS (1 μg/ml): 0.40 ± 0.037 spines/μm] and thin-type spines [Figure 5; control: 0.84 ± 0.075 spines/μm; LPS (100 ng/ml): 0.58 ± 0.039 spines/μm; LPS (1 μg/ml): 0.42 ± 0.050 spines/μm]. We found with the higher concentration of LPS, there was a further reduction in the number of thin spines resulting in a lower total spine count. As dietary DHA supplementation has been reported to suppress inflammation, we tested whether the addition of DHA in our system could prevent the damaging effects of LPS-induced inflammatory response. In slice cultures that were treated with DHA alone, we found no difference in spine density compared to control slices (Figure 5; total spine density, DHA: 1.53 ± 0.099 spines/μm, *n* = 12 cells; *p* > 0.05). More importantly, at both high and low concentrations of LPS, the addition of DHA successfully prevented the dendritic spine decrease caused by LPS treatment, as there was no difference in spine densities between the LPS + DHA-treated and control cultures [Figure 5; total spine density: LPS (1 μg/ml) + DHA: 1.72 ± 0.077 spines/μm, *n* = 16 cells; LPS (1 μg/ml) + DHA: 1.59 ± 0.083 spines/μm, *n* = 18 cells; *p* > 0.05]. It is interesting to note that even at high concentrations of LPS treatment, DHA successfully protected the damaging effects on synaptic structures caused by inflammation.

**Figure 6:**
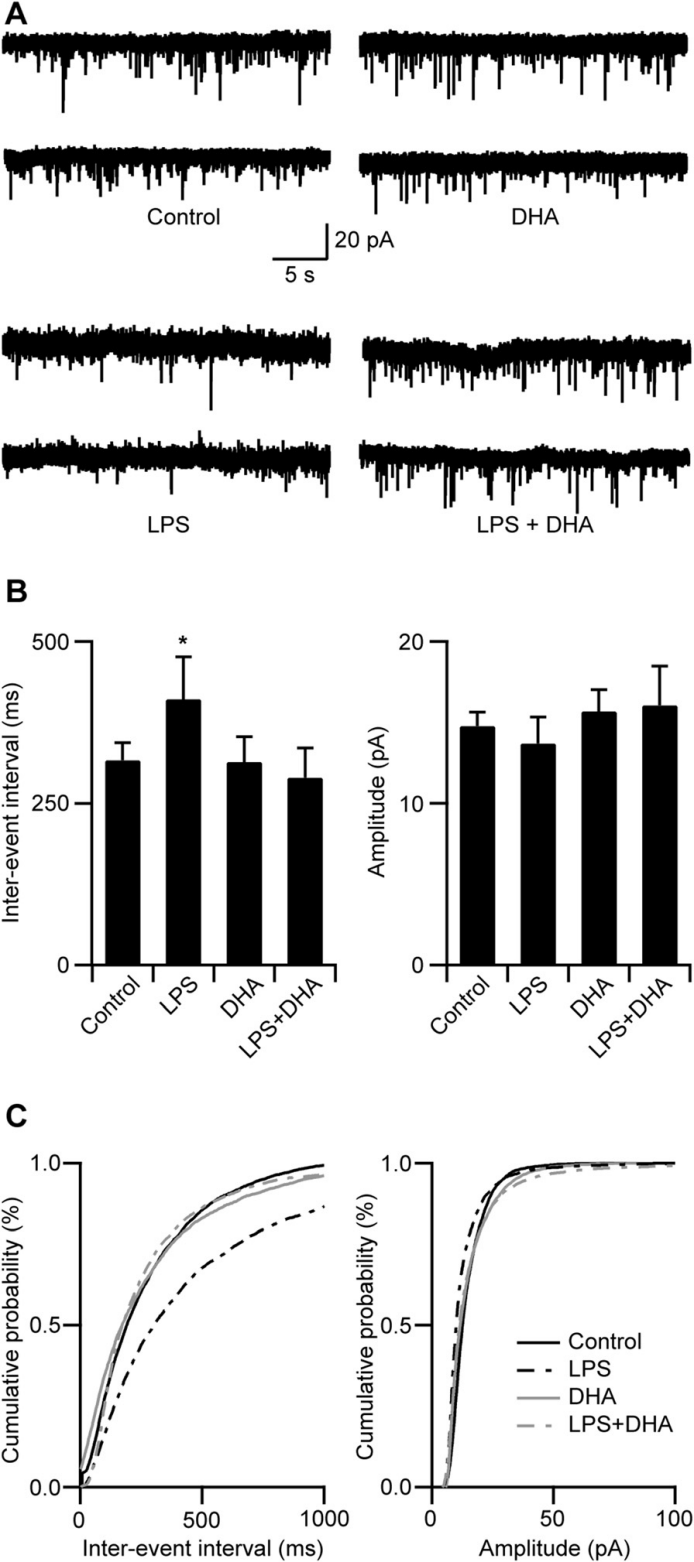
**DHA prevents LPS-induced decrease in mEPSC frequency. A** Examples of mEPSC recordings obtained from CA1 pyramidal neurons of sister cultures treated with either control, LPS, DHA or LPS + DHA-containing media. **B** Graphical representation of the averaged mEPSC IEI (*left*) and amplitude (*right*). There is a significant increase in the IEI but no changes in amplitude in the LPS-treated cultures. DHA pre-treatment prevents the decrease in IEI that is caused by LPS. * *p* < 0.05. **C** Cumulative distribution of mEPSC IEI (*left*) and amplitude (*right*) showing a significant decrease in the IEI in neurons from the LPS-treated cultures that is prevented by DHA.

**Figure 7:**
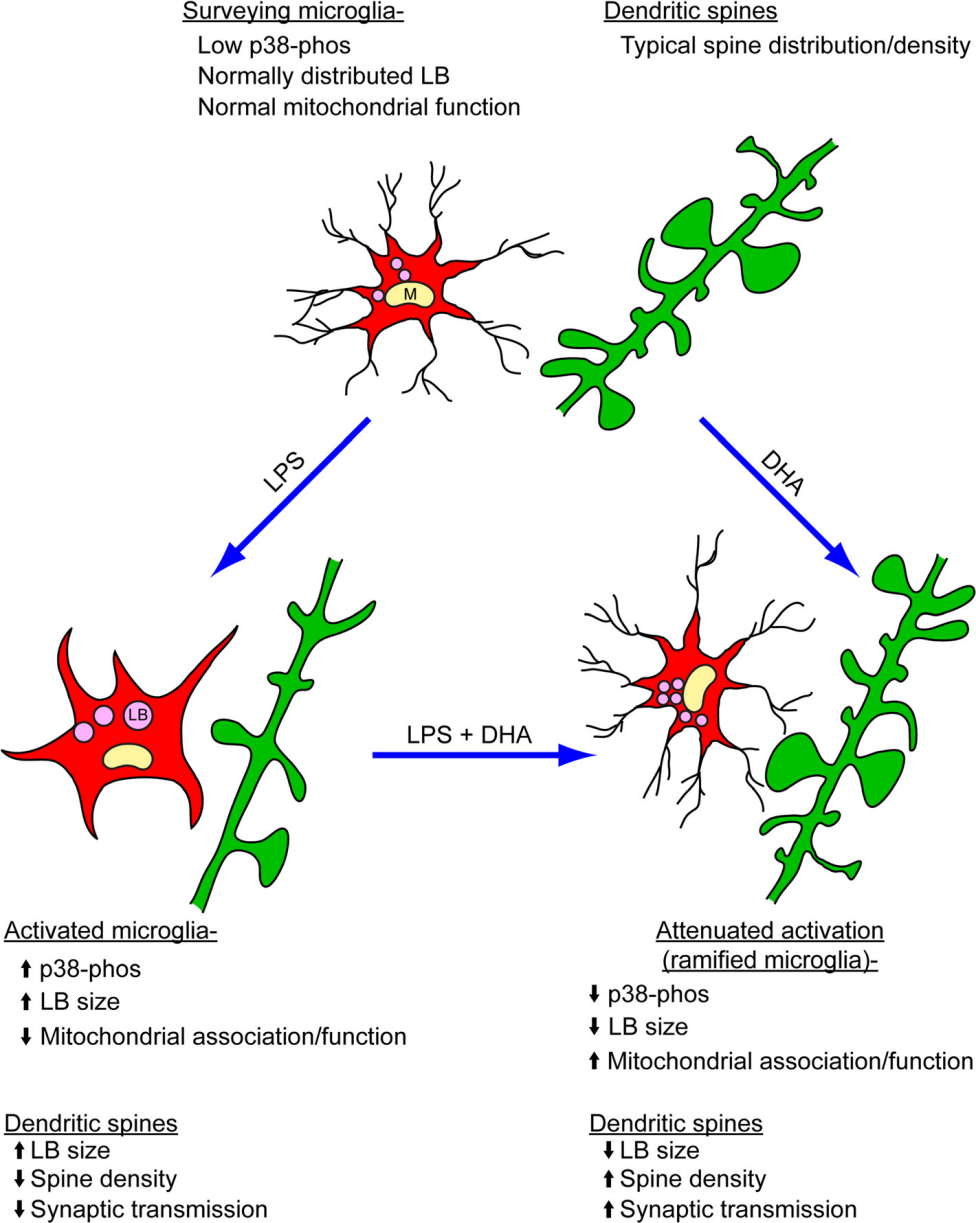
**Summarizing schematic: DHA treatment prevents microglial activation, LB aggregation and dendritic spine loss.** LPS causes microglial activation, nitric oxide release, p38 phosphorylation and increases in LB size, while it decreases dendritic spine densities in neurons. However, DHA treatment can prevent LPS-induced effects. DHA decreases nitric oxide release, p38 phosphorylation and LB size, and it restores dendritic spine densities.

As dendritic spine morphologies can influence the integrity of synaptic transmission, we examined basic synaptic transmission of the CA1 pyramidal neurons following LPS and DHA treatments in organotypic slice cultures. To assess the functional impact of spine loss, we recorded spontaneous AMPA-mediated-mEPSC in order to study the strength of the CA3-CA1 excitatory synapses. We found that with LPS (1 μg/ml) treatment, there was a significant increase in the IEI (Figure 6; control: 316.20 ± 27.08 ms, *n* = 11 cells; LPS: 410.54 ± 65.53 ms, *n* = 9 cells; *p < 0.05) with no change in mEPSC amplitude (Figure 6; control: 14.76 ± 0.86 pA; DHA: 15.68 ± 1.33 pA; LPS: 13.68 ± 1.63 pA; LPS + DHA: 16.06 ± 2.43 ms; *p* > 0.05). An increase in IEI following LPS treatment indicated that functional synapses were lost, as the occurrence of basal synaptic events had decreased. These changes in IEI, accompanied with the dendritic spine loss following LPS treatment, signified that in the LPS model of neuroinflammation, changes in the microglial activation state could negatively impact synaptic transmission. However, pre-treatment of the cultures with DHA prior to the addition of LPS successfully prevented the IEI increase associated with LPS treatment alone (Figure 6; DHA: 313.40 ± 38.89 ms, *n* = 8 cells; LPS + DHA: 289.16 ± 45.58 ms, *n* = 12 cells; *p* > 0.05). Hence, from these findings we conclude that DHA pre-treatment has the potential to prevent LPS-induced changes in microglia, including altered LB size distribution, mitochondrial function and expression of LB-associated protein levels (Figure 7). DHA prevented structural and functional synaptic alterations in CA1 pyramidal neurons induced by LPS treatment (Figure 7).

## Discussion and conclusions

The findings from this study show that LPS causes profound microglia activation in organotypic and N9 microglia cultures. This is associated with marked changes in LB number and size distribution, accompanied with changes in CA1 synaptic structure and function. Interestingly, we found that DHA treatment prior to the LPS exposure prevented microglia hyperactivity and neural impairments in CA1 in organotypic hippocampal slices. These findings are in line with an enhanced production of proinflammatory biomarkers including cytokines and a preventive effect of DHA in the brain following systemic LPS administration [49].

It is well documented that microglia can exert both positive and negative effects on neurons depending on their activation state [50]. Mildly and transiently activated microglia do not exert marked effects on dendritic spine morphologies because the numerous astrocytes within the vicinity of the neurons compensate for small imbalances between the trophic and inflammatory factors [13,51]. However, with a strong or chronic inflammatory stimulus, e.g., endotoxin LPS, the compensatory mechanisms fail in re-establishing cellular homeostasis in the brain [52-54]. As shown here, the status of activation of microglia is critical in their “synapse-sculpting” processes similar to what has been reported during neuronal circuitry development [55]. Hence, microglia hyperactivity as well as inappropriate tagging of synapses through the complement system is deleterious to synaptic structure and function [13,55-58].

In this study we have observed that exposure of hippocampal slices to high concentrations of LPS (1 μg/ml) induces loss of dendritic spines accompanied with the release of nitric oxide and formation of large LBs in neighboring microglia. LBs can be either protective or deleterious to the neuronal circuitry. We found LPS induced the formation of large LBs in microglia, which were reduced in size with DHA pretreatment prior to exposure to LPS. Large LBs formed in microglia upon LPS treatment showed only a small increase in perilipin-2. Perilipin-2 is an integral membrane protein decorating the surface of LBs that is important for the structural stability of LBs [46,59,60]. It has been shown that perilipin-2 is recruited to the LB surface and acts as a key regulator of LB stability and biogenesis [44]. The very small elevation of perilipin-2 following LPS treatment in conjunction with the marked enlargement of LB size is suggestive of instability in the phospholipid membrane of LB [61]. Such a destabilization of LBs can negatively affect lipid compartmentalization within the cell and further worsen the oxidative stress caused by LPS exposure. Free radicals such as reactive oxygen species (ROS) and reactive nitrogen species (RNS) can interact with lipids and cause lipid peroxidation to produce extremely cytotoxic compounds, which could lead to the functional impairments [62]. We found that mitochondrial function was impaired in both glia and neurons after LPS treatment, and this was accompanied by nitric oxide release and impairments in excitatory synaptic functions and structure. Our results suggest that DHA exerts protective effects in both microglia and excitatory neurons by maintaining the optimal LB size and stability by upregulation of perilipin-2 and preservation of spine morphologies. We hypothesize that the mechanisms implicated in these processes could be acting both directly on neurons and indirectly by first normalizing the biochemical changes and morphology in microglia, which then impact positively on neuronal circuitry in the hippocampal CA1 region. Although it is not fully elucidated, glial cells have the ability to produce pro-resolvin mediators, which could contribute to this positive effect [63]. In addition, DHA might modulate endocannabinoid signaling since both neurons and glia produce endocannabinoids and express endocannabinoid receptors that are implicated in neuroprotection [64].

Our studies showed that the total number of spines is preserved in CA1 pyramidal neurons in hippocampal organotypic cultures with pretreatment with DHA before LPS. DHA can directly affect the neurons through the activation of the G-protein coupled receptor, GPR120 [65,66]. DHA has also been reported to be highly enriched in synapses and plays an important role in the expression of many proteins found in the dendritic spines such as drebrin and postsynaptic density protein PSD95, both of which are essential for the integrity of excitatory synapses [67-69]. Indirect effects could be explained by DHA conversion into resolvins and protectins; these can attenuate inflammation through the downregulation of proinflammation cytokine production and an upregulation of the neurotrophin brain-derived neurotrophic factor (BDNF) [5,70-73]. It is well known that BDNF in physiological concentrations can improve synaptic circuitry [74]. In fact, it has been previously reported that elevated DHA levels in the brain can normalize BDNF levels in rat brain trauma [75]. Furthermore, the antiinflammatory effects of DHA could also be due to direct effects of DHA on the translocation of transcription factor NF-κB from the cytosol to the nucleus in microglia. Normalization NF-κB activity by DHA could also contribute to an antiinflammatory effect by attenuation of proinflammatory cytokine gene transcription driven by a nuclear pool of NF-κB (e.g., TNFα and IL-1β) [76]. Finally, DHA-induced LB increases in number, but a decrease in LB size could be favorable for LB-mitochondria interaction and preservation of mitochondrial homeostasis in microglia and neurons. Mitochondrial integrity and homeostasis are required for maintenance of synaptic circuitry [77]. A close apposition of LBs and mitochondria, as we have found in DHA-treated microglial cells, would contribute to antiinflammatory effects of DHA in different ways. First, the close association between LBs and mitochondria may facilitate the coupling of triglyceride (TG) hydrolysis and promote ATP production in mitochondria. Second, it could contribute to an enrichment of non-peroxidized cardiolipins in mitochondria [78]. Cardiolipins are lipid components that make up about 20% of the mitochondrial inner membrane, and they are involved in the regulation of mitochondrial structure and function [79-81]. A reduction of peroxidized cardiolipin species by DHA has previously been shown to reduce or even prevent mitochondrial functional impairment [82]. The promotion of mitochondrial health is in line with our observed enhancement in mitochondrial function following DHA supplementation. Future studies should employ lipidomic analysis of organelles investigated in these models to reveal both early neurodegenerative changes and beneficial effects of DHA. Ideally, lipidomic studies should be extended to the analyses of specific regions and cell types of both the central and peripheral nervous system in humans with and without DHA supplementation [83]. Thus, uncovering the mechanisms involved in the direct effects of DHA on neurons and indirectly through regulation of glia in different brain structures to preserve synaptic circuitry clearly merits further investigations [69].

## Additional file

Additional file 1: Figure S1. LPS treatment induces an upregulation in nitric oxide *release while DHA supplementation lowers* nitric oxide *release from N9 microglial cultures.* **A**, Following 24 hours of LPS treatment, there was an increase in nitric oxide secretion from N9 microglial cell cultures. **B**, Quantification of nitric oxide release from cultured microglia. There was an upregulation of in nitric oxide release following 24 hours of LPS treatment that was lowered in the presence of DHA. Control, 0.67 ± 0.12 μM; LPS, 34.33 ± 0.49 μM; DHA, 0.94 ± 0.093 μM; LPS + DHA, 22.4 ± 0.51 μM. Control, *n* = 6; LPS, *n* = 6; DHA, *n* = 5; LPS + DHA, *n* = 5; * *p* < 0.05. **Figure S2** *DHA induces p38 phosphorylation*. **A**, Example of DHA-induced increase in phosphorylated p38 levels. The effect of DHA treatment decreases in a time-dependent manner. The maximum increase in phospho-p38 was achieved following a 15-minute LPS treatment. **B**, Quantification of Western blot analysis. *n* = 6; * *p* < 0.05.

## Abbreviations

DHA: Docosahexaenoic acid
LPS: Lipopolysaccharide
mEPSCs: Miniature excitatory postsynaptic currents
LBs: Lipid bodies
CD14: Cluster of differentiation 14
TLR4: Toll-like receptor 4
PI3-K: Phosphatidylinositol 3-kinase, FOXO1, Forkhead box protein O1
JNK: c-Jun N-terminal kinase
NFκB: Nuclear factor kappa-light-chain-enhancer of activated B cells
TG: Triglycerides
PC: Phosphatidylcholine
PE: Phosphatidylethanolamine
AMPA: 2-amino-3-(3-hydroxy-5-methyl-isoxazol-4-yl) Propanoic acid
PB: Phosphate buffer
MTT: 3-(4,5-dimethylthiazol-2-yl)-2,5-Diphenyltetrazolium bromide
TTX: Tetrodotoxin
IEI: Inter-event interval
Iba-1: Ionized calcium-binding adapter molecule-1
