# Supplementary material for: Docosahexaenoic acid (DHA): a modulator of microglia activity and dendritic spine morphology
Source: J Neuroinflammation. 2015 Feb 22;12:34. doi: 10.1186/s12974-015-0244-5 (PMC4344754; doi:10.1186/s12974-015-0244-5)
Supplement: Additional file 1: Figure S1. — LPS treatment induces an upregulation in nitric oxide release while DHA supplementation lowers nitric oxide release from N9 microglial cultures. A, Following 24 hours of LPS treatment, there was an increase in nitric oxide secretion from N9 microglial cell cultures. B, Quantification of nitric oxide release from cultured microglia. There was an upregulation of in nitric oxide release following 24 hours of LPS treatment that was lowered in the presence of DHA. Control, 0.67 ± 0.12 μM; LPS, 34.33 ± 0.49 μM; DHA, 0.94 ± 0.093 μM; LPS + DHA, 22.4 ± 0.51 μM. Control, n = 6; LPS, n = 6; DHA, n = 5; LPS + DHA, n = 5; * p < 0.05. Figure S2 DHA induces p38 phosphorylation. A, Example of DHA-induced increase in phosphorylated p38 levels. The effect of DHA treatment decreases in a time-dependent manner. The maximum increase in phospho-p38 was achieved following a 15-minute LPS treatment. B, Quantification of Western blot analysis. n = 6; * p < 0.05. [file 12974_2015_244_MOESM1_ESM.pdf]

Supplemental Information:

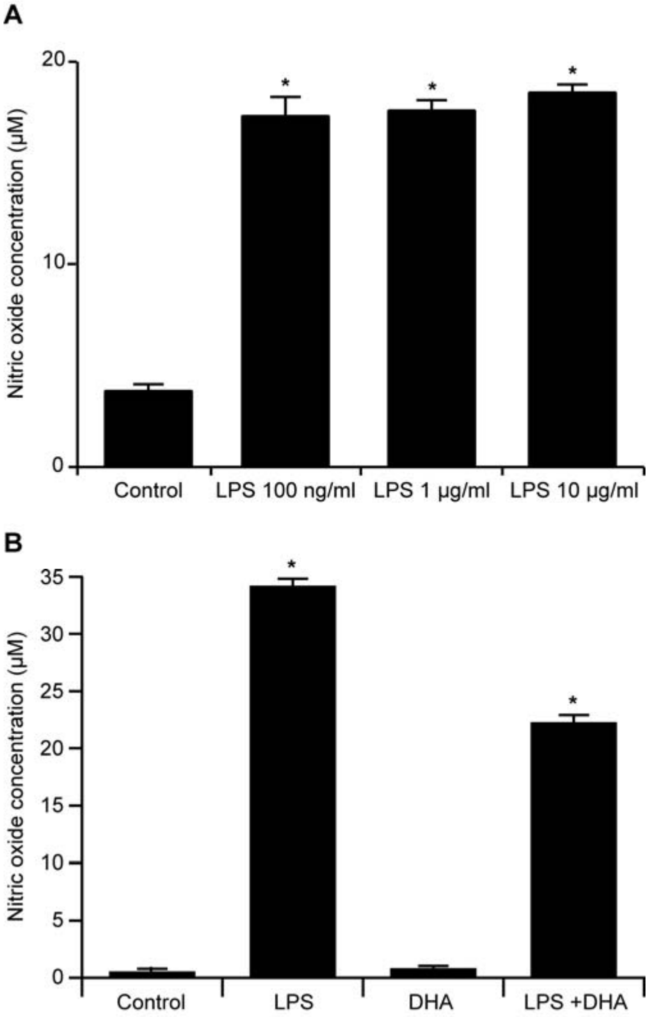

Chang et al. - Journal of Neuroinflammation - SI Figure 1

Supplemental Figure 1:

LPS treatment induces an upregulation in NO release while DHA supplementation lowers NO release from N9 microglial cultures. **A**, Following 24 hours of LPS treatment, there was an increase in NO secretion from N9 microglial cell cultures. **B**, Quantification of NO release from cultured microglia. There was an upregulation of in NO release following 24 hours of LPS treatment that was lowered in the presence of DHA. Control, 0.67±0.12 μM; LPS, 34.33±0.49 μM; DHA, 0.94±0.093 μM; LPS+DHA, 22.4±0.51 μM. Control, $n = 6$; LPS, $n = 6$; DHA, $n = 5$; LPS+DHA, $n = 5$; * $p < 0.05$.

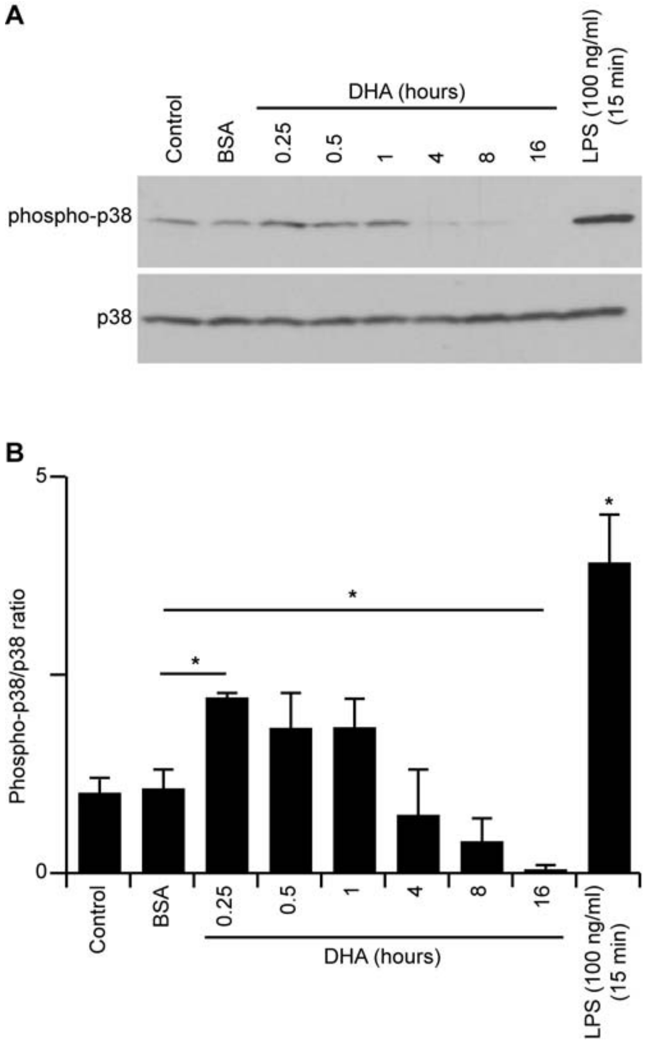

0.05.Chang et al. - Journal of Neuroinflammation - SI Figure 2

Supplemental Figure 2:

DHA induces p38 phosphorylation. **A**, Example of DHA-induced increase in phosphorylated p38 levels. The effect of DHA treatment decreases in a time-dependent manner. The maximum increase in phospho-p38 was achieved following a 15-minute LPS treatment. **B**, Quantification of Western blot analysis. $n = 6$; * $p <$
